# Efficacy and safety of ivonescimab in non-small cell lung cancer: a systematic review and meta-analysis of emerging clinical data

**DOI:** 10.3389/fphar.2026.1770637

**Published:** 2026-02-03

**Authors:** Youran Dai, Chenwei Xiao, Qi Chen, Liang Wang, Ruiqing Bo, Zerun Cheng, Guofeng Pan

**Affiliations:** 1 Department of Traditional Chinese Medicine, Beijing Shijitan Hospital affiliated to Capital Medical University, Beijing, China; 2 The First Affiliated Hospital of Zhejiang Chinese Medical University (Zhejiang Provincial Hospital of Chinese Medicine), Hangzhou, Zhejiang, China; 3 Postgraduate Affairs Department, Zhejiang Chinese Medical University, Hangzhou, Zhejiang, China; 4 Qihuang Chinese Medicine school, Beijing University of Chinese Medical, Beijing, China

**Keywords:** ivonescimab, meta-analysis, non-small cell lung cancer, PD-1, systematic review, VEGF

## Abstract

**Introduction:**

Ivonescimab (AK112), a novel bispecific antibody targeting PD-1 and VEGF, has emerged as a promising therapeutic agent in the treatment of non-small cell lung cancer (NSCLC). This study aims to comprehensively evaluate its efficacy and safety.

**Materials and methods:**

A comprehensive literature search was conducted in PubMed, Embase, Web of Science, and the Cochrane Library (from inception to January 2026) to identify studies reporting the clinical efficacy and safety outcomes of AK112 in NSCLC. Pooled analyses were conducted for efficacy endpoints, including objective response rate (ORR), disease control rate (DCR), and progression-free survival (PFS), as well as adverse events (AEs). For randomized controlled trials (RCTs), odds ratios (ORs) and hazard ratios (HRs) with 95% confidence intervals (CI) were calculated for binary and time-to-event outcomes, respectively. Subgroup analyses were performed by cancer type and treatment regimen.

**Results:**

5 studies comprising 1,365 patients were included. AK112-based regimens significantly improved ORR (OR = 1.65, 95% CI: 1.31–2.09) and DCR (OR = 2.29, 95% CI: 1.18–4.44) compared to control treatments. A significant progression in PFS was observed (HR = 0.53, 95% CI: 0.45–0.62). The PFS benefit was consistent across all PD-L1 expression subgroups. Safety analysis revealed that AK112-based regimens increased the risk of all-grade AEs (OR = 2.05, 95% CI: 1.20–3.51) and grade ≥3 AEs (OR = 1.51, 95% CI: 1.19–1.92).

**Conclusion:**

AK112 demonstrates significant efficacy and a manageable safety profile in advanced NSCLC, supporting its role as a valuable treatment option. Further studies are needed to confirm long-term survival benefits.

**Systematic Review Registration:**

https://www.crd.york.ac.uk/PROSPERO/, identifier CRD420251176434.

## 1 Introduction

Lung cancer remains one of the most common malignancies worldwide, with the highest incidence and mortality rates (George et al., 2025). Non-small cell lung cancer (NSCLC) represents the most prevalent subtype, accounting for approximately 85%–90% of all cases (Xu et al., 2023; Zhang et al., 2023). Surgical resection continues to be the most effective treatment for early-stage NSCLC (Fu et al., 2023; Pao et al., 2011). However, owing to the disease’s aggressive biology and frequent lack of early symptoms, approximately 70% of patients are diagnosed with advanced disease, rendering them ineligible for curative surgery (Huang et al., 2025; Ortega et al., 2023). Over the past decade, therapeutic options for advanced or metastatic NSCLC have expanded considerably, driven primarily by the discovery of targetable oncogenic drivers (Tan and Tan, 2022). Approximately 50% of patients with NSCLC harbor oncogenic driver mutations (Meyer et al., 2024). Among these, mutations in the epidermal growth factor receptor (EGFR) gene are the most prevalent, particularly in non-squamous NSCLC (nsq-NSCLC) and East Asian populations (Peravali et al., 2020; da Cunha Santos et al., 2011). For distinct mutation targets, multiple generations of therapeutic agents have been developed, the majority of which are tyrosine kinase inhibitors (TKIs) (Hendriks et al., 2023). In patients with NSCLC lacking actionable oncogenic mutations, immunotherapy has been integrated into standard treatment regimens.

The introduction of immunotherapy has represented another major milestone in lung cancer treatment, complementing the advances in targeted therapy (Jeon et al., 2025; Lahiri et al., 2023). Several clinical trials have demonstrated survival benefits with immune checkpoint inhibitors (ICI) in advanced NSCLC, which was considered as first-line option (Reck et al., 2022). However, primary and acquired resistance to ICIs remains a significant clinical challenge. The tumor microenvironment (TME) is a key determinant of immunotherapy response. In this context, vascular endothelial growth factor (VEGF) not only drives tumor angiogenesis but also contributes to an immunosuppressive TME (Chen et al., 2018; Harmey and Bouchier-Hayes, 2002). Agents targeting the VEGF/VEGF receptor (VEGFR) pathway have demonstrated efficacy in NSCLC (Zhao et al., 2022). Given the complementary effects of antiangiogenic agents and ICIs on TME, combining these two modalities has shown potential synergistic antitumor activity (Georganaki et al., 2018; Meder et al., 2018). These insights led to the development of ivonescimab (AK112), a bispecific humanized antibody that targets both PD-1 and VEGF-A (Dhillon, 2024). The tetravalent structure of AK112 enables the formation of large complexes with dimeric VEGF, conferring high affinity for PD-1 and enhanced functional activity, which contribute to its potent antitumor efficacy (Zhong et al., 2023). In May 2024, AK112 combined with pemetrexed and carboplatin received first approval from the U.S. Food and Drug Administration (FDA) for the treatment of patients with EGFR-mutant locally advanced or metastatic NSCLC who have experienced disease progression following TKI therapy (Dhillon, 2024). Ongoing research continues to generate clinical evidence supporting the efficacy of AK112 in broader NSCLC populations. Several studies indicate promising antitumor activity and manageable safety across patient subgroups irrespective of driver gene status. For instance, a phase 2 study confirmed significant clinical benefit from AK112 plus chemotherapy in both treatment-naïve advanced non-squamous NSCLC patients without driver mutations and EGFR-mutant patients who progressed on prior EGFR-TKI therapy (Zhao et al., 2023). Despite these promising findings, several questions remain unresolved. These include the consistency of treatment effects across different patient subgroups, the comprehensive spectrum and burden of treatment-related adverse events (AEs), and the generalizability of outcomes from controlled trials to broader clinical practice. Given the recent regulatory approval and the rapidly expanding yet fragmented clinical evidence base, a systematic synthesis of the available data is urgently needed.

In this meta-analysis, we aim to comprehensively evaluate the efficacy and safety of AK112 across various types of NSCLC, based on the emerging clinical evidence. This study intends to inform clinical decision-making and guide future research directions.

## 2 Materials and methods

This systematic review and meta-analysis were conducted in accordance with Cochrane Handbook for Systematic Reviews of Interventions and the Preferred Reporting Items for Systematic Reviews and Meta-Analyses (PRISMA) 2020 guidelines (Page et al., 2021). The protocol was registered in PROSPERO (registration number: CRD420251176434).

### 2.1 Search strategy

A comprehensive literature search was conducted across electronic databases (PubMed, Embase, Cochrane Library, Web of Science) and major conference archives, specifically those of the European Society for Medical Oncology (ESMO) and the American Society of Clinical Oncology (ASCO), for records published in English through January 2026. The search strategy used a combination of key search terms “Non-Small Cell Lung Carcinoma”, “Ivonescimab”, “AK112″and “SMT112”. The complete strategy is available in Supplementary Table S1. To ensure thorough coverage, we additionally examined the reference lists of all retrieved articles, reviews, and meta-analyses. When multiple publications reported on the same clinical trial, the publication with the most extensive and updated data was selected to prevent duplicate inclusion.

### 2.2 Eligibility criteria

Studies were included based on the following predefined criteria: 1) original articles reporting randomized controlled trials (RCTs), single-arm trials, or prospective/retrospective cohort studies; 2) patients diagnosed with NSCLC; 3) evaluated AK112 as monotherapy or part of combination therapy, with AK112 as the primary intervention; 4) reported at least one efficacy outcome (e.g., ORR, PFS) or treatment-related AEs; 5) published in English. Exclusion criteria included: 1) non-original publications (e.g., reviews, editorials, commentaries, case reports, animal studies, letter, or study protocols); 2) lacked relevant clinical outcome data; 3) duplicate or overlapping patient cohorts.

### 2.3 Quality assessment

For RCTs, the risk of bias was assessed using the Revised Cochrane Risk of Bias Tool (RoB 2.0) (Sterne et al., 2019). For non-RCTs, the Methodological Index for Non-Randomized Studies (MINORS) scale was applied (Slim et al., 2003). This instrument consists of 12 items: clearly stated aim, inclusion of consecutive patients, prospective collection of data, endpoints appropriate to the study aim, unbiased assessment of endpoints, follow-up period appropriate to the aim, loss to follow-up less than 5%, prospective calculation of sample size, adequate control group, contemporary groups, baseline equivalence of groups, and appropriate statistical analysis. Given the lack of a control group in single-arm studies, only the first eight items (maximum score = 16) were evaluated, a higher score reflects better quality. The Grading of Recommendations Assessment, Development, and Evaluation (GRADE) framework was applied to evaluate the overall certainty of evidence (Balshem et al., 2011). Summary of Findings tables were developed using GRADEpro software in accordance with Cochrane methods. The certainty of evidence for each comparison was categorized as high, moderate, low, or very low, based on assessments of study design, risk of bias, inconsistency, indirectness, imprecision, and other relevant considerations. Two investigators (YD and QC) independently performed the risk-of-bias assessments for all included studies. Any discrepancies in evaluation were resolved through consultation with a third reviewer (GP).

### 2.4 Data extraction and management

Two authors (YD and CX) independently extracted data using a standardized form. The following details were collected from each included study: first author, country, publication year, trial name, trial phase, study design, sample size, treatment, cancer stage, age, median follow-up duration, clinical endpoints, and other relevant information. Since the OS data of most studies are not yet mature, therefore, outcomes of interest included PFS, ORR, disease control rate (DCR), and the incidence of AEs. AEs, assessed as all-grade and grade ≥3, comprised neutropenia, nausea, diarrhea, anemia, neutropenia, alopecia, fatigue, leukopenia, thrombocytopenia, etc., graded according to the National Cancer Institute’s Common Terminology Criteria for Adverse Events (NCI CTCAE) (Basch et al., 2014). Any discrepancies between the reviewers were resolved through discussion with a third author during the data extraction process.

### 2.5 Statistical analysis

All statistical analyses were performed using RStudio (version 4.4.2) and Stata 17.0 (StataCorp, College Station, TX, USA). For RCTs, binary outcomes including ORR, DCR, and AEs were evaluated using odds ratios (ORs) with 95% confidence intervals (CIs). Time-to-event outcomes, including PFS, were analyzed using hazard ratios (HRs) with 95% CIs. Due to the absence of a comparator in single-arm studies, pooled proportions (P-pooled) with 95% CIs were used for ORR, DCR, and AEs. For time-to-event data, the reported median PFS (mPFS) were analysis as part of exploratory and are presented descriptively to provide supplementary insight into the efficacy of AK112. The incidence of AEs was first analyzed for all-grade and grade ≥3 events overall, and then for specific AEs categories (e.g., hematologic, gastrointestinal). Subgroup analyses were conducted based on treatment regimen, tumor type and PD-L1 expression levels. Tumor type was classified by squamous NSCLC (sq-NSCLC) and non-squamous NSCLC (nsq-NSCLC). If the number of studies is insufficient, subgroup analyses will be performed for descriptive purposes only. Both fixed-effects (Mantel-Haenszel method) and random-effects (DerSimonian-Laird method) models were utilized. Generalized linear mixed models (GLMMs) were employed in single-arm studies. Statistical heterogeneity was evaluated using Cochran’s Q test and the I^2^ statistic, with a significance level of *P* < 0.05 for the Q test or I^2^>50% indicating substantial heterogeneity. In such cases, results from the random-effects model were preferentially reported. If data allowed, we explored potential sources of statistical heterogeneity when I^2^ was above 50% through subgroup analyses on pre-defined factors. These factors include: age, prior treatment history, and duration of follow-up. Publication bias was assessed using funnel plots and Egger’s test when more than 10 studies were available. A leave-one-out sensitivity analysis was conducted on the results of the RCTs to determine the influence of possible outlier studies on the effect size. A two-sided *P* value <0.05 was considered statistically significant for all analyses.

## 3 Results

### 3.1 Search results

**FIGURE 1:**
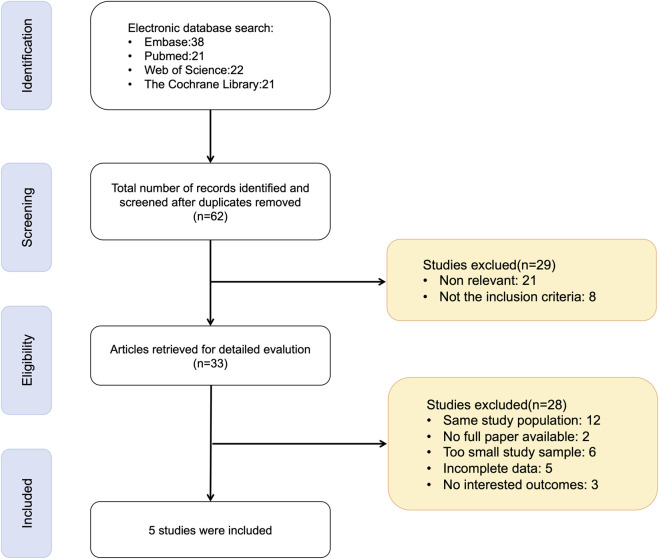
PRISMA flow diagram.

A total of 102 records were identified through systematic database searches. After removing 40 duplicates, 62 studies remained for title and abstract screening. 29 were excluded due to irrelevance, or failure to meet the inclusion criteria. The remaining 33 full-text articles were thoroughly evaluated for eligibility. Of these, 28 studies were excluded due to reasons such as small sample sizes, overlapping patient cohorts, insufficient or unavailable outcome data or incomplete reporting. Ultimately, 5 studies (Zhao et al., 2023; Chen et al., 2025a; Fang et al., 2024; Wang et al., 2024; Xiong et al., 2025) met the inclusion criteria and were included in the meta-analysis (Figure 1).

### 3.2 Basic characteristics of the included literature

This meta-analysis included 5 studies comprising a total of 1,365 patients with stage IIIB-IV NSCLC. The median age of participants ranged from 57.6 to 66 years, and the median follow-up time varied between 7.89 and 12.7 months. As one study reported data from three independent patient cohorts, these were evaluated separately in the analysis (Zhao et al., 2023). The experimental intervention consistently involved AK112, administered at doses ranging from 10 to 20 mg/kg, either as monotherapy or in combination with platinum-based chemotherapy. In the RCTs, AK112-based regimens were compared against therapies containing tislelizumab, pembrolizumab, or placebo, all combined with corresponding chemotherapy agents. The enrolled population included both treatment-naïve patients and those with prior exposure to EGFR-TKIs or systematic therapies. Commonly reported endpoints across studies comprised PFS, OS, ORR, and safety outcomes. Detailed baseline characteristics are summarized in Table 1.

**TABLE 1:** Baseline characteristics of included studies.

Author	Year	Country	Registration number	Sample size (n/%)		Cancer stage	Cancer type	Experiment			Control			Prior treatment	Median follow-up time (months)	Study type	Endpoints
				Male	Female			N	Median age (years)	Intervention	N	Median age (years)	Intervention				
ZW chen	2025	China	NCT05840016	494 (92.86)	38 (7.14)	IIIB, IIIC, IV	Squamous NSCLC	266	64.0 (59–69)	Ivonescimab 20 mg/kg + paclitaxel 175 mg/m^2^+carboplatin area under the curve [AUC] 5 mg/mL per min 4 cycles	266	64 (59–69)	Tislelizumab 200 mg + paclitaxel 175 mg/m^2^+carboplatin area under the curve [AUC] 5 mg/mL per min 4 cycles	No previous systematic therapy	10.4	RCT	PFS, OS, ORR, DCR, DOR, TTR
Aanwen Xiong	2025	China	NCT05499390	333 (83.67)	65 (16.33)	IIIB, IIIC, IV	NSCLC	198	65.0 (37–85)	Ivonescimab 20 mg/kg Q3W	200	66 (35–83)	Pembrolizumab 200 mg Q3W	No previous systematic therapy	8.7 (7.1–10.3)	RCT	PFS, OS, ORR, DOR, DCR, TTR
Lei Wang	2024	China	NCT04900363	91 (84.3)	17 (15.7)	IIIB, IIIC, IV	NSCLC	108	66.0 (48–75)	Ivonescimab 10/20/30 mg/kg Q3W/Ivonescimab 20 mg/kg Q2W	—	—	—	Systematic therapies	8.6 (0.4–16.3+)	Single trial	AES, ORR, DOR, DCR, PFS, OS
W Fang	2024	China	NCT05184712	156 (48.45)	166 (51.55)	IIIB, IIIC, IV	NSCLC	161	59.6 (32.3–74.9)	Ivonescimab 20 mg/kg + pemetrexed 500 mg/m^2^ Q3W	161	59.4 (36.2–74.2)	Placebo plus pemetrexed 500 mg/m2 Q3W	PD after EGFR-TKI therapy	7.89	RCT	PFS, OS, ORR, DOR, DCR, TTR
Yuanyuan Zhao	2023	China	NCT04736823	34 (77.8)	10 (22.2)	Advanced	NSCLC	44	57.6 (44.3–73.0)	AK112 (10 or 20 mg/kg) combined with pemetrexed (500 mg/m^2^ ) for non- sqNSCLC or paclitaxel (175 mg/m2) for sq-NSCLC+ carboplatin (area under the curve of 5 mg/mL per min), four cycles	—	—	—	No previous systematic therapy	12.7 (11.6–13.8)	Single trial	ORR, AES, PFS, DCR, DOR, OS
Yuanyuan Zhao	2023	China	NCT04736823	6 (31.6)	13 (68.4)	Advanced	NSCLC	19	60.2 (34.7–64.9)	AK112 (10 or 20 mg/kg) plus pemetrexed plus carboplatin, 4 cycles	—	—	—	EGFR-tyrosine kinase inhibitor (TKI) therapy	11.0 (8.5–12.9)	Single trial	ORR, AES, PFS, DCR, DOR, OS
Yuanyuan Zhao	2023	China	NCT04736823	16 (80.0)	4 (20.2)	Advanced	NSCLC	20	60.0 (31.6–73.4)	AK112 (10 or 20 mg/kg) plus docetaxel (75 mg/m^2^)	—	—	—	Systematic therapies	10.3 (8.1–11.7)	Single trial	ORR, AES, PFS, DCR, DOR, OS

NSCLC, non‐small cell lung cancer; RCT, randomized controlled trial; PD, progressive disease; EGFR, epidermal growth factor receptor; PFS, progression‐free survival; OS, overall survival; ORR, objective response rate; DCR, disease control rate; DOR, duration of response; TTR, time to response; AES, adverse events.

### 3.3 Quality assessment

The quality assessment of 3 RCTs and two single-arm studies was well reported. For RCTs, the overall risk of bias was judged to be at low risk (Supplementary Figure S1). Two single-arm studies were included, both of which were rated as having moderate methodological quality. The MINORS assessment details for each study are provided in Supplementary Table S2.

### 3.4 Efficacy

#### 3.4.1 Objective response rate (ORR)

**FIGURE 2:**
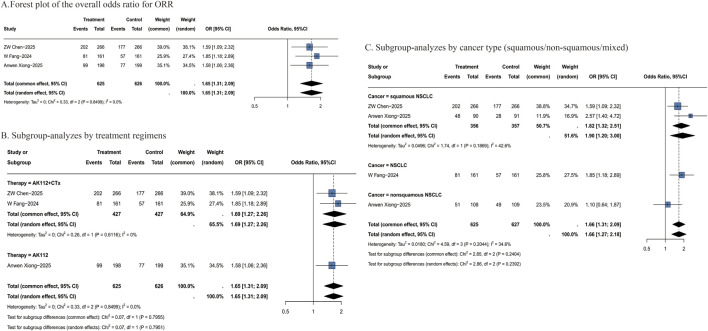
Forest plots of ORR. **(A)** Forest plot of the overall odds ratio for ORR; **(B)** Subgroup-analyzes by treatment regimens; **(C)** Subgroup-analyzes by cancer type (squamous/non-squamous/mixed). ORR: objective response rate; OR: odds ratio; CI: confidence interval; P-pooled: pooled proportion; N: number of patients; Events: number of responders.

All studies reported ORR outcomes. Treatment with AK112-based regimens was associated with a significantly higher objective response rate compared to control treatments (OR = 1.65, 95% CI: 1.31–2.09; Figure 2A). This treatment effect was further enhanced in patients receiving AK112 combined with chemotherapy (OR = 1.69, 95% CI: 1.27–2.26; Figure 2B) and was particularly pronounced in the sq-NSCLC subgroup (OR = 1.90, 95% CI: 1.20–3.00; Figure 2C). Given the limited number of included RCTs and the variability in treatment regimens, further analyses of specific treatment combinations are presented as descriptive summaries (Supplementary Table S3). Among these, “20 mg/kg AK112 Q3W + pemetrexed + carboplatin” regimen showed promising potential (OR = 1.85, 95% CI: 1.18–2.89).

Data from single-arm studies provided supportive evidence, with a pooled ORR of 52% (95% CI: 44%–60%; Supplementary Figure S2). Higher response rates were observed in both the “10 mg/kg AK112 Q3W + pemetrexed + carboplatin” subgroup (80%) and the sq-NSCLC subgroup (58%). Overall, current evidence suggests that certain AK112-based combination regimens, particularly in conjunction with chemotherapy, may demonstrate enhanced therapeutic potential. Furthermore, promising efficacy signals were observed specifically in patients with sq-NSCLC. The detailed results are presented in Supplementary Table S4. However, these conclusions still require further validation through more prospective RCTs.

#### 3.4.2 Progression free survival (PFS)

**FIGURE 3:**
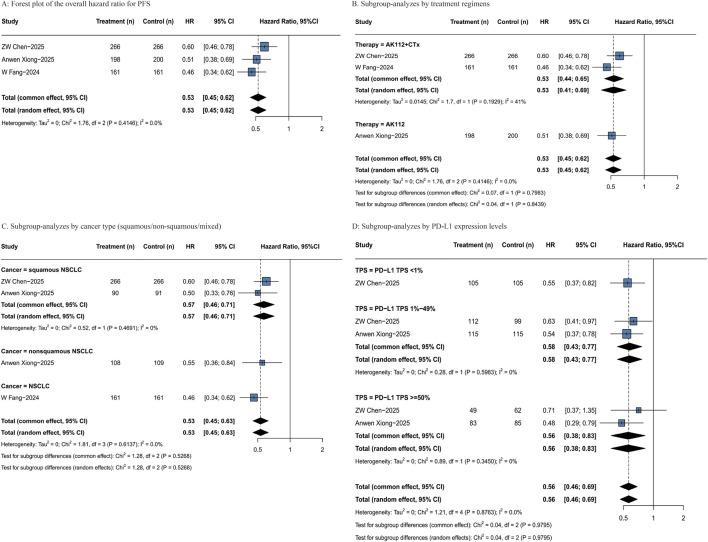
Forest plots of PFS. **(A)** Forest plot of the overall hazard ratio for PFS; **(B)** Subgroup-analyzes by treatment regimens; **(C)** Subgroup-analyzes by cancer type (squamous/non-squamous/mixed).; **(D)** Subgroup-analyzes by PD-L1 expression levels. PFS: progression-free survival; HR: hazard ratio; mPFS: median progression-free survival; CI: confidence interval; N: number of patients; Events: number of responders.

All RCTs reported PFS outcomes, involving a total of 1,252 patients. AK112 can significantly reduce the risk of disease progression or death compared to conventional chemotherapy (HR = 0.53, 95% CI: 0.45–0.62; Figure 3A). Subgroup analyses revealed consistent PFS benefits across key patient populations, the treatment effect remained robust and comparable between major therapeutic and cancer type (Figures 3B,C). A descriptive summary of outcomes by specific treatment regimen is provided in Supplementary Table S3, which shows that the “20 mg/kg AK112 Q3W + pemetrexed + carboplatin” regimen was associated with a particularly pronounced risk reduction (HR = 0.46, 95% CI: 0.34–0.62). Further analysis confirmed that AK112 treatment provided significant PFS improvement across all Programmed cell Death protein-1 (PD-L1) expression levels, with HR of 0.55 (95% CI: 0.37–0.82) in TPS <1%, 0.58 (95% CI: 0.43–0.77) in TPS 1%–49%, and 0.56 (95% CI: 0.38–0.83) in TPS ≥50% subgroups (Figure 3D). Supplementary Table S3 provides a further description of the patient population based on the treatment regimens.

Results from the two single-arm studies are reported descriptively and were not pooled for analysis due to limitations in survival data maturity. In the study by Zhao et al. (2023), survival data were immature. Although Lei Wang et al. reported a median PFS of 11.4 months, there was a lack of mature subgroup data, preventing further evaluation.

#### 3.4.3 Disease control rate (DCR)

**FIGURE 4:**
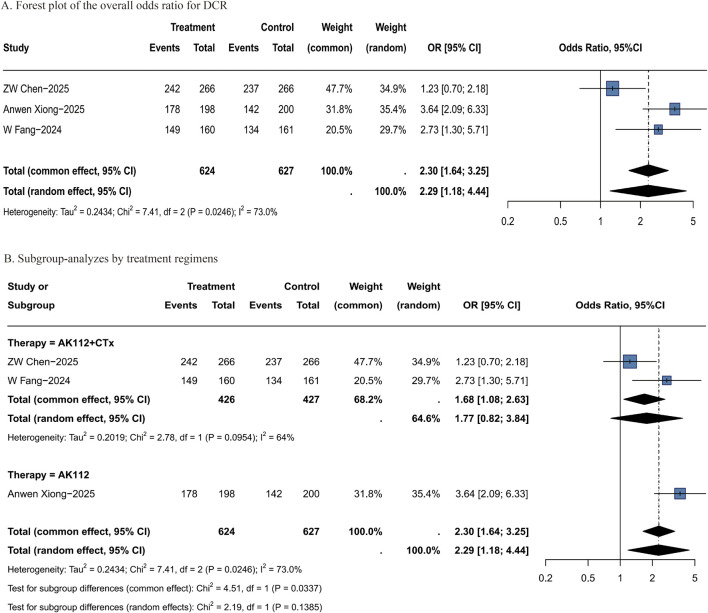
Forest plots of DCR. **(A)** Forest plot of the overall odds ratio for DCR; **(B)** Subgroup-analyzes by treatment regimens. DCR: disease control rate; OR: odds ratio; CI: confidence interval; P-pooled: pooled proportion; N: number of patients; Events: number of responders.

Analysis of RCT data indicated that AK112-based regimens significantly improved DCR compared to conventional chemotherapy (OR = 2.29, 95% CI: 1.18–4.44; Figure 4A), with the AK112 monotherapy subgroup showing a more pronounced benefit (OR = 3.64, 95% CI: 2.09–6.33; Figure 4B). Outcomes by treatment regimen are shown in Supplementary Table S3. Among these, “20 mg/kg AK112 Q3W” regimen may have more advantages (OR = 3.64, 95% CI: 2.09–6.33). Due to limited data availability on tumor subtypes, subgroup analysis by histologic type was not performed. The outcomes stratified by specific treatment regimens are presented in Supplementary Table S3.

These findings were further supported by data from single-arm studies, which reported a pooled DCR of 88% (95% CI: 82%–92%; Supplementary Figure S3) for AK112 treatment. Subgroup analysis by regimen indicated a DCR of 93% (95% CI: 68%–100%) for “20 mg/kg AK112 Q3W” (Supplementary Table S4). However, these results should be interpreted with caution given the wide confidence intervals approaching 100% and the limited sample size.

### 3.5 Safety

#### 3.5.1 Main safety analyses

**FIGURE 5:**
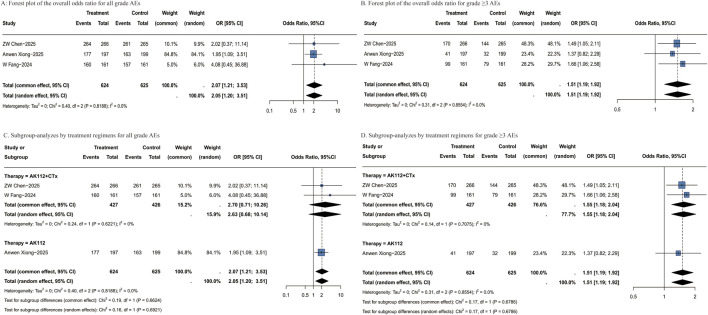
Forest plots of AEs. **(A)** Forest plot of the overall odds ratio for all grade AEs; **(B)** Forest plot of the overall odds ratio for grade ≥3 AEs; **(C)** Subgroup-analyzes by treatment regimens for all grade AEs; **(D)** Subgroup-analyzes by treatment regimens for grade ≥3 AEs. AEs: adverse events; OR: odds ratio; CI: confidence interval; P-pooled: pooled proportion; N: number of patients; Events: number of responders.

Safety data from all included RCTs were systematically analyzed. AK112-based regimens were associated with a significantly increased risk of all-grade AEs compared to control treatments (OR = 2.05, 95% CI: 1.20–3.51; Figure 5A). The risk of grade ≥3 AEs remained significantly elevated in the AK112 group (OR = 1.51, 95% CI: 1.19–1.92; Figure 5B). Subgroup analysis by treatment demonstrated that AK112 combined with chemotherapy was associated with an increased risk of all-grade AEs (OR = 2.70, 95% CI: 0.71–10.26; Figure 5C), though this result did not reach statistical significance. Similarly, for grade ≥3 adverse events, the combination therapy subgroup showed an OR of 1.55 (95% CI: 1.18–2.04; Figure 5D). Given that only one RCT reported outcomes separately for patients with sq-NSCLC, no further subgroup analysis based on histologic tumor type was performed. More detailed subgroups stratified by treatment regimen are provided in Supplementary Table S3.

The pooled data from single-arm studies showed a very high incidence of all-grade AEs at 99% (95% CI: 96%–100%; Supplementary Figure S4A), with the AK112-chemotherapy combination reaching 100%. For grade ≥3 AEs, the overall incidence was 44% (95% CI: 35%–54%), and 52% (95% CI: 41%–63%) for the combination (Supplementary Figure S4B). Subgroup analysis showed a 100% of all-grade AE incidence in sq-NSCLC and a higher grade ≥3 AE incidence (40%) in nsq-NSCLC. The 30 mg/kg AK112 Q3W was associated with the lowest AE rates (all-grade: 85%; grade ≥3: 15%). Due to the limited sample size and the wide CI (which in most cases included 100%), these results should be interpreted as exploratory and treated with caution (Supplementary Table S4).

#### 3.5.2 Specific safety analyses

We synthesized safety data reported in both single-arm and RCTs to preliminarily characterize the adverse event profile of AK112 based regimens (Table 2,3). Given the limited number of available studies and the heterogeneity in data sources (single-arm and RCT designs), a formal meta-analysis was not performed. The following results represent only descriptive summaries of incidence rates. In AK112 combination chemotherapy regimens, hematologic toxicities predominated. The incidence rates of any-grade anemia, neutropenia, and thrombocytopenia were 56.6%, 48.9%, and 31.6%, respectively. Alopecia was also common, with an incidence of 55.2%. Grade ≥3 neutropenia occurred at a rate of 25.5%, representing the primary dose-limiting toxicity. In contrast, AK112 monotherapy was associated with rare hematologic toxicities. Non-hematologic adverse events constituted the principal safety signals in this setting, such as Proteinuria (24.21% in all grade AEs) and hypertension (13.29% in all grade AEs). Meanwhile, the overall incidence of serious AEs (grade ≥3) was relatively low. Due to the limited number of studies and data heterogeneity, this analysis remains exploratory and descriptive, and the conclusions should be interpreted with caution.

**TABLE 2:** Pooled incidence of all-grade adverse events.

Category	Adverse events	Combintion		Single	
		N of study	Incidence	N of study	Incidence
Hematological toxicity	Anemia	3	56.62%	2	12.10%
	Neutropenia	3	48.93%	—	—
	Thrombocytopenia	3	31.62%	—	—
	Leukopenia	3	32.91%	—	—
	Lymphopenia	2	10.62%	—	—
Hepatotoxicity	ALT increased	3	26.07%	2	14.09%
	AST increased	3	23.72%	2	17.46%
	GGT increased	3	13.35%	1	11.11%
	Bilirubin increased	1	6.02%	2	11.71%
	ALP increased	1	11.10%	—	—
Metabolic and endocrine disorders	Hyperglycemia	1	15.53%	2	12.70%
	Hypercholesterolemia	2	13.63%	2	16.27%
	Hyperlipidemia	2	7.74%	1	10.19%
	Hypothyroidism	2	9.61%	2	11.90%
	Hyperthyroidism	1	4.52%	1	3.79%
	TSH increased	1	3.61%	1	1.77%
Renal and urinary toxicity	Proteinuria	3	18.70%	2	24.21%
	Albuminuria	2	4.72%	—	—
	Hypoalbuminemia	2	15.73%	1	11.36%
	BUN increased	1	9.60%	1	17.59%
Cardiovascular toxicity	Hypertension	2	7.82%	2	13.29%
	ATE	1	0.94%	2	0.51%
Immune-related adverse events	Immune-mediated lung disease	1	2.64%	1	3.28%
Gastrointestinal toxicity	Diarrhea	2	7.33%	—	—
	Nausea	2	15.80%	—	—
	Vomiting	2	24.20%	—	—
	Constipation	3	15.92%	—	—
	Decreased appetite	2	27.20%	—	—
General and constitutional symptoms	Fatigue	2	15.73%	—	—
	Weight loss	3	10.90%	—	—
	Pain	3	12.63%	—	—
	Alopecia	2	55.21%	—	—
	Rash	3	9.94%	1	10.86%
	Pruritus	1	8.43%	1	12.04%
Hemorrhagic events	Haemorrhage	1	15.44%	1	12.88%
	Hemoptysis	2	7.98%	—	—
Other laboratory abnormalities	LDH increased	2	13.09%	1	10.19%
	CRP increased	1	7.23%	1	23.15%
	AMY increased	2	12.84%	2	8.73%

N, number; ALT, alanine aminotransferase; AST, aspartate aminotransferase; GGT, gamma‐glutamyl transferase; ALP, alkaline phosphatase; TSH, thyroid‐stimulating hormone; BUN, blood urea nitrogen; ATE, arterial thromboembolic events; LDH, lactate dehydrogenase; CRP, C‐reactive protein; AMY, amylase.

**TABLE 3:** Pooled incidence of grade ≥3 adverse events.

Category	Adverse events	Combination		Single	
		N of study	Incidence	N of study	Incidence
Hematological toxicity	Anemia	3	8.44%	1	1.01%
	Neutropenia	3	25.53%	—	—
	Thrombocytopenia	3	6.73%	—	—
	Leukopenia	3	12.93%	—	—
	Lymphopenia	2	4.94%	—	—
Hepatotoxicity	ALT increased	2	1.29%	1	0.51%
	AST increased	2	1.06%	2	0.40%
	GGT increased	3	1.60%	1	0.93%
Metabolic and endocrine disorders	Hyperglycaemia	1	0.62%	1	0.76%
	Hypertriglyceridemia	1	1.69%	1	1.26%
Renal and urinary toxicity	Proteinuria	1	0.82%	2	1.39%
Cardiovascular toxicity	Hypertension	1	2.07%	2	2.38%
	ATE	1	0.56%	1	0.51%
Immune-related adverse events	Immune-mediated lung disease	1	1.32%	1	1.26%
Gastrointestinal toxicity	Diarrhea	2	0.65%	—	—
General and constitutional symptoms	Weight loss	2	0.47%	—	—
	Pain	3	0.61%	—	—
	Rash	2	1.17%	1	0.25%
Hemorrhagic events	Haemorrhage	1	1.32%	1	0.76%
	Haemoptysis	2	0.98%	—	—
Other laboratory abnormalities	AMY increased	2	0.74%	1	0.76%

N, number; ALT, alanine aminotransferase; AST, aspartate aminotransferase; GGT, gamma‐glutamyl transferase; ALP, alkaline phosphatase; TSH, thyroid‐stimulating hormone; ATE, arterial thromboembolic events; AMY, amylase.

### 3.6 Subgroup analyses

Based on the results of RCTs, significant heterogeneity was observed in the DCR outcome (I^2^ = 73%). To explore potential sources of this heterogeneity, we conducted a subgroup analysis of all outcomes reported in the RCTs, based on age, prior treatment history, and duration of follow-up. The analysis revealed a statistically significant difference in DCR across subgroups defined by follow-up duration (*P* = 0.008), while all other outcomes remained consistent across the subgroups. These findings suggest that variation in follow-up duration may contribute to the observed heterogeneity in DCR. Detailed results are presented in Table 4. For single-arm studies, given that only two trials were included, no further exploratory subgroup analysis was performed, and no significant heterogeneity was identified in the available data.

**TABLE 4:** Subgroup analysis for RCTs.

Subgroups	Categories	ORR					PFS					DCR					AEs (all grade)					AEs (grade ≥3)				
		N	OR	95%CI	I^2^	*P*	N	HR	95%CI	I^2^	*P*	N	OR	95%CI	I^2^	*P*	N	OR	95%CI	I^2^	*P*	N	OR	95%CI	I^2^	*P*
Time	≤10 m	2	1.70	1.27–2.29	0.0%	0.773	2	0.48	0.39–0.60	0%	0.216	2	3.28	2.11–5.11	0.0%	0.008	2	2.07	1.18–3.65	0.0%	0.988	2	1.53	1.09–2.14	0.0%	0.915
	>10 m	1	1.59	1.09–2.32	—	—	1	0.60	0.46–0.78	0%	​	1	1.23	0.70–2.18	—	—	1	2.02	0.37–11.14	—	—	1	1.49	1.05–2.11	—	—
Prior	No previous systematic therapy	2	1.59	1.21–2.10	0.0%	0.578	2	0.56	0.46–0.68	0%	0.290	2	2.12	0.74–6.12	85.9%	0.703	2	1.96	1.13–3.42	0.0%	0.528	2	1.45	1.09–1.93	0.0%	0.620
	PD after EGFR-TKI therapy	1	1.85	1.18–2.89	—	—	1	0.46	0.34–0.62	0%	—	1	2.73	1.30–5.71	—	—	1	4.08	0.45–36.88	—	—	1	1.56	1.06–2.58	—	—
Age	≤60y	1	1.85	1.18–2.89	—	0.578	1	0.46	0.34–0.62	—	0.290	1	2.73	1.30–5.71	—	0.703	1	4.08	0.45–36.88	—	0.528	1	1.66	1.06–2.58	—	0.620
	>60y	2	1.59	1.21–2.10	0.0%	—	2	0.56	0.46–0.68	0%	—	2	2.12	0.74–6.12	85.9%	—	2	1.96	1.13–3.42	0.0%	—	2	1.45	1.09–1.93	0.0%	—

OR: odds ratio; HR: hazard ratio; CI: confidence interval; N: number of studies; ORR: objective response rate; PFS: progression-free survival; DCR: disease control rate; AEs: adverse events; PD: progressive disease; EGFR-TKI: epidermal growth factor receptor-tyrosine kinase inhibitor.

### 3.7 Sensitivity analysis and publication bias

A leave-one-out sensitivity analysis was performed for the four outcomes of the RCTs (Supplementary Figure S5). However, due to the small number of included studies, only an exploratory analysis was conducted. The pooled effect estimates for ORR, AES (all-grade and grade ≥3), and PFS remained consistent in direction after the sequential exclusion of each individual study, suggesting the robustness of these results. In contrast, for DCR, the OR showed a substantial variation after the exclusion of the study by ZW Chen et al. (Chen et al., 2025a), indicating relatively limited robustness of this outcome. Given the small number of included studies (n < 10), no assessment of publication bias was conducted.

### 3.8 Certainty of evidence

The overall findings of the GRADE assessment are summarized in Supplementary Table S5. The certainty of evidence for all outcomes was downgraded by one level due to potential publication bias associated with the limited number of included studies. The DCR outcome in the combination therapy group was rated as very low certainty, primarily due to inconsistency and imprecision. The evidence for any-grade AEs in the combination therapy group and for grade ≥3 AEs in the monotherapy group was rated as low certainty, owing to imprecision.

## 4 Discussion

The treatment landscape for NSCLC has been transformed by targeted therapies and immunotherapies. Despite these advances, significant challenges remain, including the nearly inevitable development of acquired resistance to EGFR-TKIs and the limited response rates observed with ICI monotherapy in a substantial proportion of patients. To address this therapeutic impasse, AK112, a novel bispecific antibody targeting PD-1 and VEGF, was developed. We present the first systematic review and meta-analysis to comprehensively evaluate the clinical efficacy and safety of AK112. Our results demonstrate that AK112-based regimens significantly improve ORR and prolong PFS compared with conventional chemotherapy. Notably, the PFS benefit was consistent across all PD-L1 expression subgroups, offering a promising therapeutic strategy particularly for the difficult-to-treat PD-L1 negative population. Furthermore, while the incidence of all-grade AEs was high, the safety profile of AK112 was manageable overall. These results collectively underscore the potential of this novel PD-1/VEGF bispecific antibody to address key limitations in the current NSCLC treatment landscape.

The significant efficacy and favorable safety profile of AK112 are attributable to its innovative molecular design and dual mechanism of action. Approved by the U.S. FDA under accelerated assessment in May 2024 (Dhillon, 2024) and by the China NMPA in the same month, AK112 is indicated for EGFR mutated nsq-NSCLC following EGFR TKI therapy failure (Cui et al., 2021). It was included in China’s National Reimbursement Drug List in November 2024 (Nikoo et al., 2023) and is currently under global evaluation for multiple solid tumors. As a humanized IgG1-scFv bispecific antibody, AK112 simultaneously targets PD-1 and VEGF in the tumor microenvironment (Zhong et al., 2025). This coordinated blockade of both immune checkpoint and angiogenic pathways promotes T cell infiltration and enhances antitumor immunity (Cui et al., 2021; Shi et al., 2025). Our results revealed that the combination of AK112 and chemotherapy is associated with improved antitumor activity. Mechanistically, chemotherapy-mediated immunogenic cell death may have expanded the range of tumor-specific T cells (Galluzzi et al., 2017), meanwhile, AK112 can foster a permissive microenvironment for T-cell infiltration and cytotoxicity (Wu et al., 2016; Caicun Zhou et al., 2022). This combined action may promote a more immunogenic and less immunosuppressive tumor state. Our subgroup analysis also demonstrating consistent PFS benefit across all PD-L1 expression subgroups, including patients with TPS below 1%, 1%–49%, and 50% or higher, with HR ranging from 0.55 to 0.58. These results suggest that AK112’s dual targeting strategy may overcome the limitation of PD-L1 dependency, offering an effective therapeutic approach for patients with PD-L1 negative or low expression tumors who typically respond poorly to immune checkpoint inhibitor monotherapy. The underlying mechanism may involve simultaneous reversal of immune suppression and improvement of tumor vascular function, collectively remodeling the tumor microenvironment to overcome resistance to conventional immunotherapy (Zhang et al., 2017; Zhong et al., 2023). In summary, these findings position AK112, both as a monotherapy and particularly in combination with chemotherapy, as a promising therapeutic strategy that expands treatment options and overcomes key limitations of existing immunotherapies.

Our study also identified differential efficacy of AK112 between sqand nsq-NSCLC. sq-NSCLC may achieve a better ORR (OR: 1.90; 95% CI: 1.20–3.00), and the results from the single-arm study also confirmed this. However, no significant difference was observed in PFS between squamous and non-squamous subgroups, indicating that AK112 significantly reduces the risk of disease progression across different histological types. This discrepancy may stem from distinct molecular characteristics between the two histological subtypes. Studies indicate that while approximately 50% of nsq-NSCLC harbor targetable driver genes (e.g., EGFR, ALK, ROS1), sq-NSCLC typically lacks these actionable alterations despite its complex genetics, leading to fewer treatment options and a generally poorer prognosis (Tan et al., 2020). Evidence from clinical trials further supports the consistent benefit of AK112 across histological types (Chen et al., 2025a; Xiong et al., 2025). These findings demonstrate that AK112 may offer a new treatment option for squamous NSCLC patients who have limited therapeutic choices. However, it should be noted that the limited number of included studies precluded more detailed subgroup analyses based on molecular profiling and other biomarkers. These findings indicate that when evaluating the efficacy of AK112 in clinical practice, comprehensive consideration of histological type, PD-L1 expression status, and driver gene mutation profile is essential to more accurately identify the optimal patient population for treatment. Additionally, the therapeutic potential of AK112 may extend beyond NSCLC, as early-phase trials suggest activity in small cell lung cancer (SCLC) (Chen et al., 2025b), though this warrants further investigation.

Regarding safety, our meta-analysis confirmed that AK112-based regimens are associated with a significantly increased risk of both all-grade (OR = 2.05) and grade ≥3 adverse events (OR = 1.51) compared to control treatments. These findings were consistent across sensitivity analyses, supporting the robustness of the conclusion. Pooled data from single-arm studies indicated a very high incidence of all-grade AEs (99%), which was nearly universal in combination therapy subgroups. However, the incidence of severe (grade ≥3) events was lower, suggesting that while AEs are common, most are clinically manageable. The safety profile differed meaningfully between monotherapy and combination regimens. Hematologic toxicities, particularly neutropenia, anemia, and thrombocytopenia, were the predominant and most severe toxicities in combination therapy. This safety profile aligns with expectations for carboplatin-based chemotherapy, which formed the backbone of combination regimens. Carboplatin is one of the most widely used chemotherapeutic agents, with hematologic toxicity and severe myelosuppression being its primary dose-limiting toxicities (Doshi et al., 2012; Oun et al., 2018). The main manifestations of carboplatin-induced myelosuppression include thrombocytopenia and neutropenia (Groopman and Itri, 1999; Marani et al., 1996). Thus, the occurrence of AEs in the AK112 combination regimen may be due to the toxicity of chemotherapy drugs. The underlying mechanisms involve multi-level pathophysiological processes. Upon entering cells, carboplatin forms platinum-DNA adducts that trigger DNA double-strand breaks (Chen et al., 2017). When the repair capacity of bone marrow hematopoietic cells is insufficient, the p53-mediated apoptotic pathway is activated, leading to a reduction in hematopoietic cell numbers. Concurrently, carboplatin exacerbates this process by disrupting the hematopoietic microenvironment, inhibiting the secretion of key cytokines such as stem cell factor and IL-3 from bone marrow stromal cells, thereby impairing their supportive function for hematopoietic cells (Dygai et al., 2007). These interconnected mechanisms collectively contribute to the development of myelosuppression, primarily manifested as thrombocytopenia and neutropenia. Compared to its modest hematological toxicity in monotherapy, AK112 exhibits a distinct profile of non-hematological adverse events. As a PD-1/VEGF bispecific agent, it notably presents a low incidence of immune-related adverse events. This may be attributed to the Fc-silencing L234A/L235A mutations engineered within the IgG-scFv structural framework of AK112 (Zhong et al., 2023). This engineered design eliminates Fc-mediated effector functions, including antibody-dependent cellular cytotoxicity, thereby substantially reducing the risk of immune-related toxicity. As for VEGF target, AK112 exhibits class-related adverse events similar to other VEGF inhibitors, notably proteinuria and hypertension. Proteinuria arises mainly from disrupted glomerular filtration due to VEGF-VEGFR-2 blockade in podocytes, while sustained inhibition may promote glomerular thrombotic microangiopathy (Estrada et al., 2019; Izzedine et al., 2010). Hypertension is linked to downstream effects of VEGF inhibition, including reduced nitric oxide, elevated endothelin-1, and microvascular rarefaction (Robinson et al., 2010; Kappers et al., 2010; Baffert et al., 2006). In our study, monotherapy showed slightly higher rates of proteinuria and hypertension than combination therapy with chemotherapy, though bleeding and thrombotic events did not differ significantly, which indicating no additive VEGF toxicity from combination treatment. Thus, blood pressure and urinary protein should be monitored clinically (Rashidi et al., 2023). However, the interpretation of the overall adverse event profile requires caution due to the limited number of studies. Overall, AK112 demonstrates a manageable safety profile.

The subgroup analyses provided important insights into potential sources of heterogeneity. Significant statistical heterogeneity was observed in the DCR outcome across RCTs (I^2^ = 73%). Exploration of pre-specified factors indicated that follow-up duration was a significant modifier of the DCR effect (*P* = 0.008), with a more pronounced benefit observed in studies with shorter follow-up (≤10 months) compared to the single trial with longer follow-up (>10 months). This pattern should be interpreted cautiously, as it likely reflects the limited number of available studies and the relative immaturity of the current evidence base for this novel agent, rather than a definitive time-dependent attenuation of treatment effect. Importantly, long-term follow-up is critical for fully evaluating outcomes in cancer therapy, as it allows for the assessment of response durability and late-onset safety signals (SEER Training Modules: Importance of Cancer Patient Follow-Up, 2025). Nevertheless, the current finding underscores the potential influence of assessment timing on endpoint interpretation and highlights the importance of standardizing outcome assessment timelines in future trials to improve comparability. Further studies with longer follow-up and larger sample sizes are warranted to better characterize the durability of disease control with AK112 and to validate these preliminary observations.

This study has several strengths. To our knowledge, it is the first meta-analysis to comprehensively evaluate the efficacy and safety of AK112 in NSCLC. We first conducted a meta-analysis of RCTs and supplemented the findings by integrating data from single-arm studies. This approach enabled the construction of a comprehensive evidence synthesis framework. We not only synthesized key efficacy endpoints, but also systematically assessed the incidence of all-grade and grade ≥3 AEs, with detailed categorization of specific event types. Furthermore, subgroup analyses were conducted across multiple clinically relevant factors. These analyses revealed differences in the efficacy and safety of AK112 across various patient populations, which may offer valuable insights for individualized clinical treatment strategies. However, several limitations should be considered. First, as OS data were largely immature or unreported in the included studies, our analysis primarily relied on PFS and ORR as the main efficacy endpoint. Although these also serves as an important surrogate marker, improvement in OS remains the ultimate indicator of patient benefit and the key goal of clinical treatment. Therefore, the impact of AK112-containing regimens on OS warrants further validation in future studies. Second, the number of studies included in the analysis was limited, only five trials were included. This also contributed to the lower certainty in the GRADE assessment. Although subgroup analyses were performed to investigate the sources of substantial heterogeneity, the small number of included studies still constitutes a potential source of instability. Third, all included studies were conducted in China, which may limit the generalizability of our findings to other ethnic and geographic populations. Therefore, international multi-center studies are warranted in the future to validate these findings in broader, more diverse populations. Moreover, it should be noted that, in analyzing the detailed spectrum of AEs, a pooled descriptive analysis was conducted incorporating data from both RCTs and single-arm studies. This combined approach precludes formal comparative safety assessments between the experimental and control groups and should therefore be regarded as a methodological limitation. Future research should prioritize large-scale, multi-regional RCTs with long-term follow-up to confirm the survival benefits, safety profiles, and establish the optimal positioning of AK112 within the evolving NSCLC treatment paradigm.

## 5 Conclusion

This meta-analysis demonstrates that AK112 significantly improves ORR, PFS and DCR in patients with NSCLC. The treatment benefits were consistent across different PD-L1 expression levels and histological subtypes. The safety profile of AK112 was generally manageable, with hematologic toxicities representing the most common adverse events, particularly when combined with chemotherapy. These findings establish AK112 as a promising therapeutic option for NSCLC patients, especially those with limited treatment alternatives. Further studies with longer follow-up durations and larger patient cohorts are warranted to validate the long-term survival benefits and refine patient selection criteria.

## Data Availability

The original contributions presented in the study are included in the article/Supplementary Material, further inquiries can be directed to the corresponding author.
