# Supplementary material for: Efficacy and safety of ivonescimab in non-small cell lung cancer: a systematic review and meta-analysis of emerging clinical data
Source: Front Pharmacol. 2026 Feb 3;17:1770637. doi: 10.3389/fphar.2026.1770637 (PMC12909539; doi:10.3389/fphar.2026.1770637)
Supplement: Supplementary file 1 [file DataSheet1.pdf]

Efficacy and safety of Ivonescimab in non-small cell lung cancer: a systemic review and meta-analysis of emerging clinical data

Supplemental data

Supplementary Table S1. Search strategies.

Supplementary Table S2. Risk of bias assessment for single-arm studies.

Supplementary Table S3. Efficacy and safety outcomes between different treatment regimens in RCTs.

Supplementary Table S4. Pooled safety outcomes between different subgroup in single-arm studies.

Supplementary Table S5. GRADE evidence assessment.

Supplementary Figure S1. Risk of bias assessment results of RCTs.

Supplementary Figure S2. Pooled results of ORR for single-arm studies.

Supplementary Figure S3. Pooled results of DCR for single-arm studies.

Supplementary Figure S4. Pooled results of AEs for single-arm studies.

Supplementary Figure S5. Sentivity analysis results.

Supplementary Table S1. Search strategies.

**Pubmed**	
**#1**	Carcinoma, Non-Small-Cell Lung[MeSH Terms]
**#2**	(Carcinoma, Non Small Cell Lung[Title/Abstract]) OR (Carcinomas, Non-Small-Cell Lung[Title/Abstract]) OR (Lung Carcinoma, Non-Small-Cell[Title/Abstract]) OR (Lung Carcinomas, Non-Small-Cell[Title/Abstract]) OR (Non-Small-Cell Lung Carcinomas[Title/Abstract]) OR (Non-Small-Cell Lung Carcinoma[Title/Abstract]) OR (Non Small Cell Lung Carcinoma[Title/Abstract]) OR (Carcinoma, Non-Small Cell Lung[Title/Abstract]) OR (Non-Small Cell Lung Carcinoma[Title/Abstract]) OR (Non-Small Cell Lung Cancer[Title/Abstract]) OR (Non small Cell Lung Cancer[Title/Abstract])
**#3**	#1 OR #2
**#4**	"Ivonescimab[Title/Abstract] OR AK112[Title/Abstract] OR SMT112[Title/Abstract]"
**#5**	"clinical"[Title/Abstract] OR "trial"[Title/Abstract] OR "clinical trial"[Title/Abstract] OR "randomized controlled trial"[Title/Abstract] OR "cohort"[Title/Abstract] OR "prospective"[Title/Abstract] OR "retrospective"[Title/Abstract] (clinical [Title/Abstract]) OR (trial[Title/Abstract]) OR (clinical trial[Title/Abstract]) OR (randomized controlled trial[Title/Abstract]) OR (RCT[Title/Abstract]) OR (cohort[Title/Abstract]) OR (prospective[Title/Abstract]) OR (retrospective[Title/Abstract]) OR (observational[Title/Abstract]) OR (control group[Title/Abstract])
**#6**	#3 AND #4 AND #5
**Embase**	
**#1**	'non small cell lung cancer'/exp
**#2**	'carcinoma, non small cell lung':ti,ab,kw OR 'carcinomas, non-small-cell lung':ti,ab,kw OR 'lung carcinoma, non-small-cell':ti,ab,kw OR 'lung carcinomas, non-small-cell':ti,ab,kw OR 'non-small-cell lung carcinomas':ti,ab,kw OR 'non-small-cell lung carcinoma':ti,ab,kw OR 'non small cell lung carcinoma':ti,ab,kw OR 'carcinoma, non-small cell lung':ti,ab,kw OR 'non-small cell lung carcinoma':ti,ab,kw OR 'non-small cell lung cancer':ti,ab,kw OR 'nonsmall cell lung cancer':ti,ab,kw
**#3**	#1 OR #2
**#4**	'Ivonescimab':ti,ab,kw OR 'AK112':ti,ab,kw OR 'SMT112':ti,ab,kw
**#5**	'Randomized':ti,ab,kw OR 'Randomised':ti,ab,kw OR 'RCT':ti,ab,kw OR 'RCTs':ti,ab,kw OR 'control group':ti,ab,kw OR

	'placebo':ti,ab,kw OR 'cohort':ti,ab,kw OR 'prospective':ti,ab,kw OR 'retrospective':ti,ab,kw OR 'observational':ti,ab,kw
**#6**	#3 AND #4 AND #5
**Cochrane Library**	
**#1**	MeSH descriptor: [Carcinoma, Non-Small-Cell Lung] explode all trees
**#2**	(Carcinoma, Non Small Cell Lung OR Carcinomas, Non-Small-Cell Lung OR Lung Carcinoma, Non-Small-Cell OR Lung Carcinomas, Non-Small-Cell OR Non-Small-Cell Lung Carcinomas OR Non-Small-Cell Lung Carcinoma OR Non Small Cell Lung Carcinoma OR Carcinoma, Non-Small Cell Lung OR Non-Small Cell Lung Carcinoma OR Non-Small Cell Lung Cancer OR Nonsmall Cell Lung Cancer):ti,ab,kw
**#3**	#1 OR #2
**#4**	'Ivonescimab':ti,ab,kw OR 'AK112':ti,ab,kw OR 'SMT112':ti,ab,kw
**#5**	(Randomized OR Randomised OR RCT OR RCTs OR control group OR placebo OR cohort OR prospective OR retrospective OR observational):ti,ab,kw
**#6**	#3 AND #4 AND #5
**Web of science**	
**#1**	TS=(Carcinoma, Non-Small-Cell Lung OR Carcinomas, Non-Small-Cell Lung OR Lung Carcinoma, Non-Small-Cell OR Lung Carcinomas, Non-Small-Cell OR Non-Small-Cell Lung Carcinomas OR Non-Small-Cell Lung Carcinoma OR Non Small Cell Lung Carcinoma OR Carcinoma, Non-Small Cell Lung OR Non-Small Cell Lung Carcinoma OR Non-Small Cell Lung Cancer OR Non small Cell Lung Cancer)
**#2**	TS=(Ivonescimab OR AK112 OR SMT112)
**#3**	TS=(clinical OR trial OR clinical trial OR randomized controlled trial OR cohort OR prospective OR retrospective OR observational OR control group)
**#4**	#1 AND #2 AND #3

Supplementary Table S2. Risk of bias assessment for single-arm studies.

Study	A clearly stated aim	Inclusion of consecutive patients	Prospective collection of data	Endpoints appropriate to the aim	Unbiased assessment of the study endpoint	Follow-up period appropriate to the aim	Loss to follow up less than 5%	Prospective calculation of the study size	Total score
Wang et al. 2024 (HARMONI-5)	2	2	2	2	2	1	1	1	13/16
Zhao et al. 2023	2	1	2	2	2	2	2	1	14/16

Supplementary Table S3. Efficacy and safety outcomes between different treatment regimens in RCTs.

Study	N(tretament)	N(Control)	Therapy	ORR			PFS			DCR			AES (all grade)			AES (grade ≥3)		
				OR (95%CI)	I^2	*P*	HR (95%CI)	I^2	*P*	OR (95%CI)	I^2	*P*	OR (95%CI)	I^2	*P*	OR (95%CI)	I^2	*P*
ZW Chen−2025	266	266	20 mg/kg AK112 Q3w+P+C	1.59 (1.09-2.32)	0.0%	0.857	0.60 (0.46-0.78)	73%	0.025	1.23 (0.70-2.18)	0.0%	0.857	2.02 (0.37-11.14)	0.0%	0.819	1.49 (1.05-2.10)	0.0%	0.856
Anwen Xiong−2025	198	200	20 mg/kg AK112 Q3w	1.60 (1.07-2.38)	/	/	0.51 (0.38-0.69)	/	/	3.64 (2.09-6.33)	/	/	1.95 (1.09-3.51)	/	/	1.37 (0.82-2.29)	/	/
W Fang−2024	161	161	20 mg/kg AK112 Q3w+Pem+C	1.85 (1.18-2.89)	/	/	0.46 (0.34-0.62)	/	/	2.73 (1.30-5.71)	/	/	4.08 (0.45-36.88)	/	/	1.66 (1.06-2.58)	/	/

N: number of patients; ORR: objective response rate; PFS: progression-free survival; DCR: disease control rate; AEs: adverse events; OR: odds ratio; HR: hazard ratio; CI: confidence interval; AK112: Ivonescimab; Q3w: every 3 weeks; P: paclitaxel; Pem: pemetrexed; C: carboplatin.

Supplementary Table S4. Pooled safety outcomes between different subgroup in single-arm studies

A. Pooled efficacy outcomes between different treatment regimens.

Study	N	Therapy	ORR			DCR		
			P-pooled (95%CI)	I^2	*P*	P-pooled (95%CI)	I^2	*P*
Lei Wang−2024	21	10mg/kg AK112 Q3w	33% (15%-57%)	46.4%	/	86% (64%-97%)	0.0%	/
Lei Wang−2024	19	20mg/kg AK112 Q2w	47% (24%-71%)	/	/	84% (60%-97%)	/	/
Lei Wang−2024	15	20mg/kg AK112 Q3w	60% (32%-84%)	/	/	93% (68%-100%)	/	/
Lei Wang−2024	12	30mg/kg AK112 Q3w	75% (43%-95%)	/	/	92% (62%-100%)	/	/
Yuanyuan Zhao−2023	19	10 mg/kg AK112 Q3W+Pem/P+C	53% (29%-76%)	0.0%	/	89% (67%-99%)	8.7%	/
Yuanyuan Zhao−2023	24	20 mg/kg AK112 Q3W+Pem/P+C	54% (33%-74%)	/	/	96% (79%-100%)	/	/
Yuanyuan Zhao−2023	10	10 mg/kg AK112 Q3W+Pem+C	80% (44%-97%)	/	/	90% (55%-100%)	/	/
Yuanyuan Zhao−2023	9	20 mg/kg AK112 Q3W+Pem+C	56% (21%-86%)	/	/	100% (66%-100%)	/	/
Yuanyuan Zhao−2023	10	10 mg/kg AK112 Q3W+D	40% (12%-74%)	/	/	70% (35%-93%)	/	/
Yuanyuan Zhao−2023	10	20 mg/kg AK112 Q3W+D	40% (12%-74%)	/	/	70% (35%-93%)	/	/

B. Pooled efficacy outcomes between different cancer type.

Study	N	cancer type	P-pooled (95%CI)		
			ORR	AES (all grade)	AES (≥3)
Lei Wang−2024	55	squamous NSCLC	58% (44%-71%)	100% (94%-100%)	36% (24%-50%)
	53	non-squamous NSCLC	58% (44%-72%)	96% (87%-100%)	40% (26%-54%)

N: number of patients; ORR: objective response rate; PFS: progression-free survival; DCR: disease control rate; P-pooled: pooled proportion; mPFS: median progression-free survival; CI: confidence interval; AK112: Ivonescimab; Q2w: every 2 weeks; Q3w: every 3 weeks; P: paclitaxel; Pem: pemetrexed; C: carboplatin; D: docetaxel.

Supplementary Table S5. GRADE evidence assessment

ORR											
	**Quality assessment**							**No. of patients**		**Effect**	**Quality**
**Comparison**	**No. of studies**	**Design**	**Risk of bias**	**Inconsistency**	**Indirectness**	**Imprecision**	**Other considerations**	**Intervention-1**	**Intervention-2**	**OR (95% CI)**	
**AK112+CTx vs CTx**	2	randomised trials	no serious	no serious	no serious	no serious	suspected	427	427	1.69 (1.27-2.26)	⊕⊕⊕O MODERATE
**AK112 vs CTx**	1	randomised trials	no serious risk of bias	no serious inconsistency	no serious indirectness	no serious imprecision	suspected	196	199	1.58 (1.06-2.36)	⊕⊕⊕O MODERATE
**PFS**											
	**Quality assessment**							**No. of patients**		**Effect**	**Quality**
**Comparison**	**No. of studies**	**Design**	**Risk of bias**	**Inconsistency**	**Indirectness**	**Imprecision**	**Other considerations**	**Intervention-1**	**Intervention-2**	**HR (95% CI)**	
**AK112+CTx vs CTx**	2	randomised trials	no serious risk of bias	no serious inconsistency	no serious indirectness	no serious imprecision	suspected	427	427	0.53 (0.41-0.69)	⊕⊕⊕O MODERATE
**AK112 vs CTx**	1	randomised trials	no serious risk of bias	no serious inconsistency	no serious indirectness	no serious imprecision	suspected	198	200	0.51 (0.38-0.69)	⊕⊕⊕O MODERATE
**DCR**											
	**Quality assessment**							**No. of patients**		**Effect**	**Quality**
**Comparison**	**No. of studies**	**Design**	**Risk of bias**	**Inconsistency**	**Indirectness**	**Imprecision**	**Other considerations**	**Intervention-1**	**Intervention-2**	**OR (95% CI)**	

**AK112+CTx vs CTx**	2	randomised trials	no serious risk of bias	serious	no serious indirectness	serious	suspected	426	427	1.77 (0.82-3.84)	⊕OOO VERY LOW
**AK112 vs CTx**	1	randomised trials	no serious risk of bias	no serious inconsistency	no serious indirectness	no serious imprecision	suspected	198	200	3.64 (2.09-6.33)	⊕⊕⊕O MODERATE
**AES (all grade)**											
	**Quality assessment**							**No. of patients**		**Effect**	**Quality**
**Comparison**	**No. of studies**	**Design**	**Risk of bias**	**Inconsistency**	**Indirectness**	**Imprecision**	**Other considerations**	**Intervention-1**	**Intervention-2**	**SMD (95% CI)**	
**AK112+CTx vs CTx**	2	randomised trials	no serious risk of bias	no serious inconsistency	no serious indirectness	serious	suspected	427	427	2.70 (0.71-10.26)	⊕⊕OO LOW
**AK112 vs CTx**	1	randomised trials	no serious risk of bias	no serious inconsistency	no serious indirectness	no serious imprecision	suspected	196	199	1.95 (1.09-3.51)	⊕⊕⊕O MODERATE
**AES (grade≥3)**											
	**Quality assessment**							**No. of patients**		**Effect**	**Quality**
**Comparison**	**No. of studies**	**Design**	**Risk of bias**	**Inconsistency**	**Indirectness**	**Imprecision**	**Other considerations**	**Intervention-1**	**Intervention-2**	**SMD (95% CI)**	
**AK112+CTx vs CTx**	2	randomised trials	no serious risk of bias	no serious inconsistency	no serious indirectness	no serious imprecision	suspected	427	427	1.55 (1.18-2.04)	⊕⊕⊕O MODERATE
**AK112 vs CTx**	1	randomised trials	no serious risk of bias	no serious inconsistency	no serious indirectness	serious	suspected	196	199	1.37 (0.82-2.29)	⊕⊕OO LOW

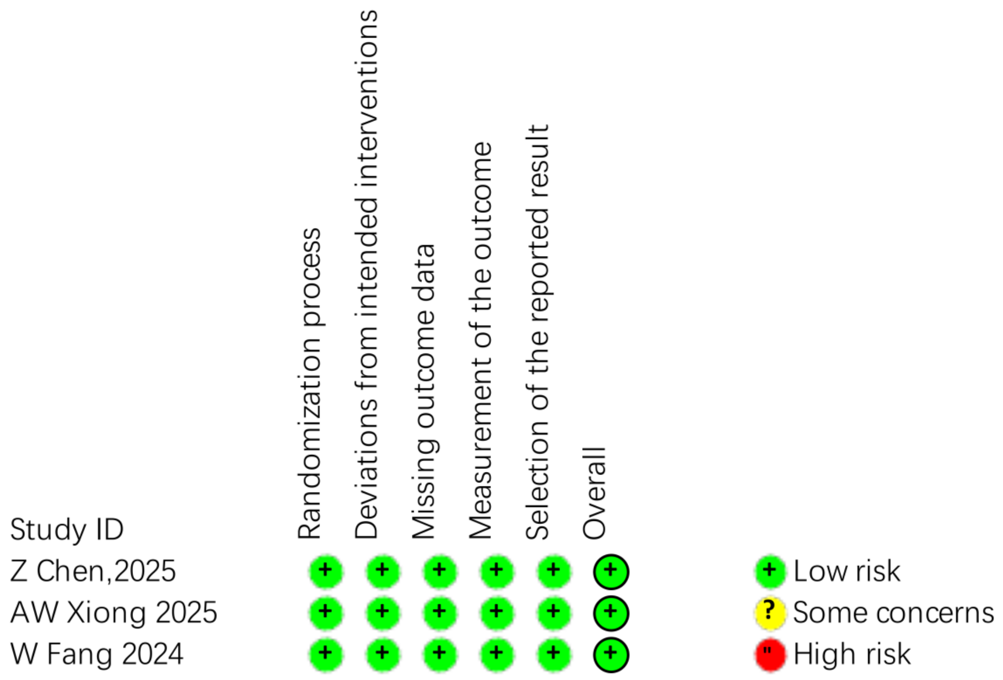

Supplementary Figure S1. Risk of bias assessment results of RCTs.
RCT: randomized controlled trial

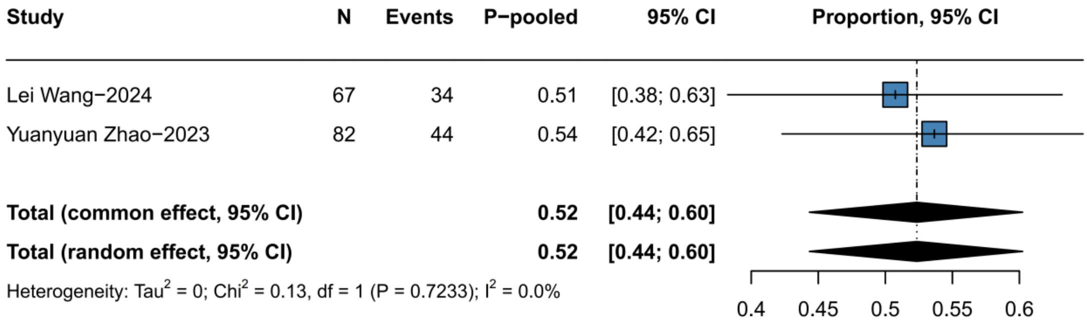

Supplementary Figure S2. Pooled results of ORR for single-arm studies.

ORR: objective response rate; P-pooled: pooled proportion; CI: confidence interval; N: number of patients; Events: number of responders.

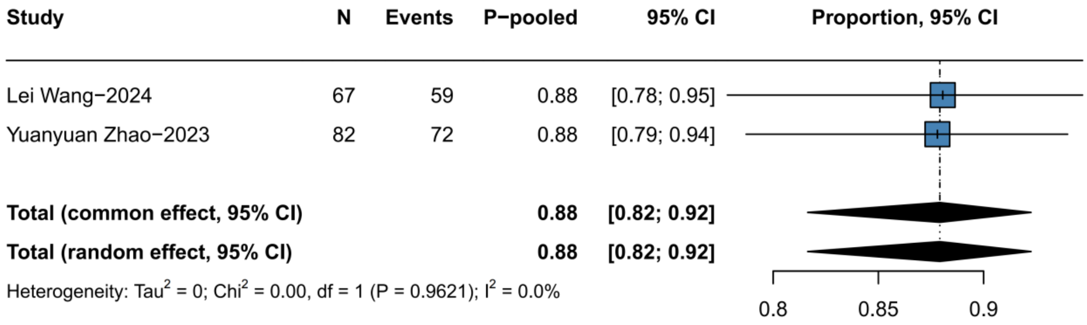

Supplementary Figure S3. Pooled results of DCR for single-arm studies.

DCR: disease control rate; P-pooled: pooled proportion; CI: confidence interval; N: number of patients; Events: number of responders.

A

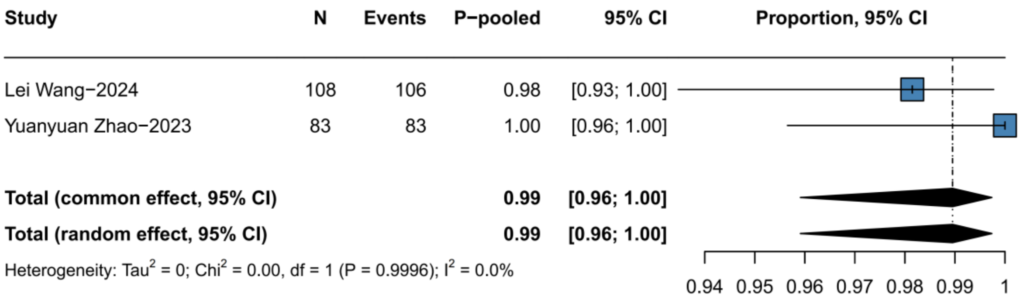

B

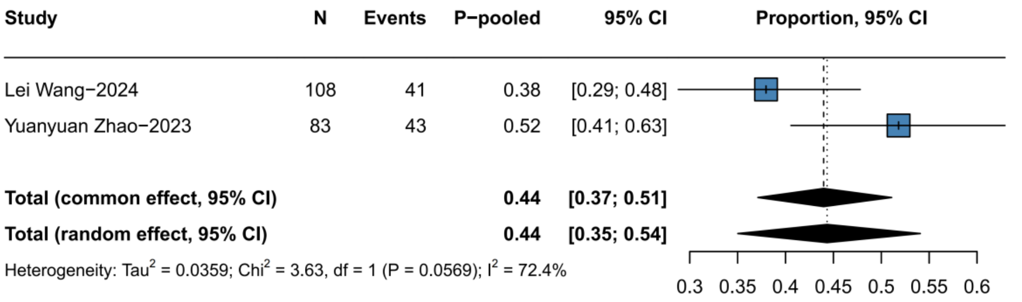

Supplementary Figure S4. Pooled results of AEs for single-arm studies.

A: Pooled result for all grade AEs; B: Pooled result for grade ≥3 AEs.

AEs: adverse events; P-pooled: pooled proportion; CI: confidence interval; N: number of patients; Events: number of responders.

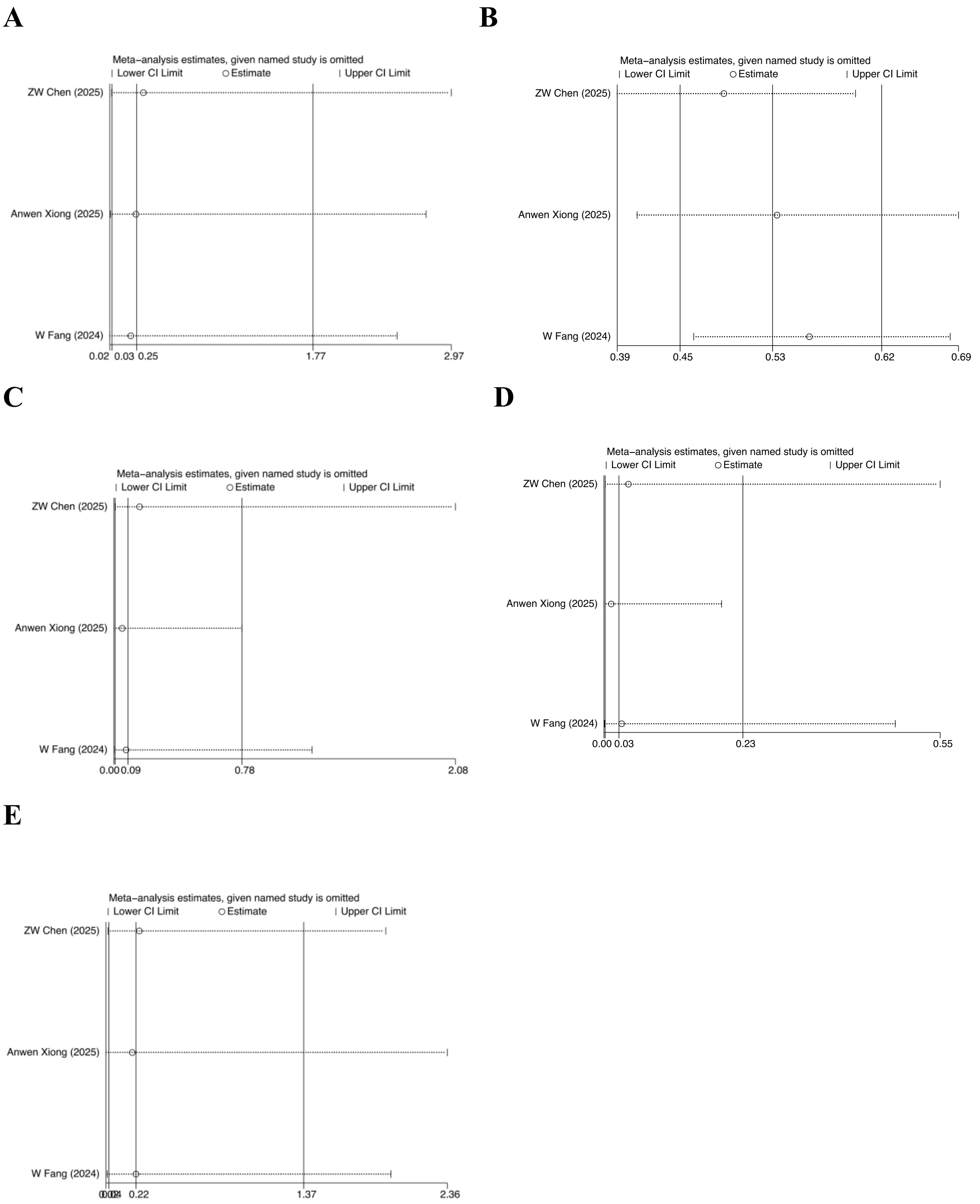

Supplementary Figure S5. Sentivity analysis results.

A: Sentivity analysis for ORR; B: Sentivity analysis for PFS; C: Sentivity analysis for DCR; D: Sentivity analysis for all grade AEs; E: Sentivity analysis for grade ≥3 AEs. ORR: objective response rate; PFS: progression fress survival; DCR: disease control rate; AEs: adverse events.
